# Large database for the analysis and prediction of spliced and non-spliced peptide generation by proteasomes

**DOI:** 10.1038/s41597-020-0487-6

**Published:** 2020-05-15

**Authors:** Gerd Specht, Hanna P. Roetschke, Artem Mansurkhodzhaev, Petra Henklein, Kathrin Textoris-Taube, Henning Urlaub, Michele Mishto, Juliane Liepe

**Affiliations:** 1 Max-Planck-Institute for Biophysical Chemistry, 37077 Göttingen, Germany; 2 Charité – Universitätsmedizin Berlin, corporate member of Freie Universität Berlin, Humboldt-Universität zu Berlin, and Berlin Institute of Health, Institute of Biochemistry, D-10117 Berlin, Germany; 3 Charité – Universitätsmedizin Berlin, corporate member of Freie Universität Berlin, Humboldt-Universität zu Berlin, and Berlin Institute of Health, Shared Facility for Mass Spectrometry, D-10117 Berlin, Germany; 4 Centre for Inflammation Biology and Cancer Immunology (CIBCI) & Peter Gorer Department of Immunobiology, King’s College London, SE1 1UL London, United Kingdom

**Keywords:** Peptides, Proteases, Proteomic analysis

## Abstract

Proteasomes are the main producers of antigenic peptides presented to CD8^+^ T cells. They can cut proteins and release their fragments or recombine non-contiguous fragments thereby generating novel sequences, *i.e*. spliced peptides. Understanding which are the driving forces and the sequence preferences of both reactions can streamline target discovery in immunotherapies against cancer, infection and autoimmunity. Here, we present a large database of spliced and non-spliced peptides generated by proteasomes *in vitro*, which is available as simple CSV file and as a MySQL database. To generate the database, we performed *in vitro* digestions of 55 unique synthetic polypeptide substrates with different proteasome isoforms and experimental conditions. We measured the samples using three mass spectrometers, filtered and validated putative peptides, identified 22,333 peptide product sequences (15,028 spliced and 7,305 non-spliced product sequences). Our database and datasets have been deposited to the Mendeley (doi:10.17632/nr7cs764rc.1) and PRIDE (PXD016782) repositories. We anticipate that this unique database can be a valuable source for predictors of proteasome-catalyzed peptide hydrolysis and splicing, with various future translational applications.

## Abstract (summary)

**Table:** 

Measurement(s)	peptide
Technology Type(s)	mass spectrometry
Factor Type(s)	spliced/non-spliced • instrument • synthetic polypeptide • proteasome isoform • time of reaction
Sample Characteristic - Organism	Homo sapiens

Machine-accessible metadata file describing the reported data: 10.6084/m9.figshare.12205274

## Background & Summary

Proteasomes are likely the most important proteases in eukaryotic cells. They destroy transcription factors, obsolete, damaged, wrongly transcribed proteins. Often the peptide fragments derived from this process are further trimmed by aminopeptidases to provide the amino acids needed for new protein synthesis. In some cases, their released protein/peptide fragments are actively involved in metabolic and immunological pathways. For instance, proteasomes can cleave p105, thereby generating a component of the transcription factor NF- κB, and osteopontin, thereby releasing peptides that promote cell migration^1–3^. The most famous “active” peptide products released by proteasomes, however, are antigenic peptides bound to Human Leukocyte Antigen class I (HLA-I) complexes (*i.e*. the HLA-I immunopeptidome) and presented at the cell surface to CD8^+^ T cells^4^.

Eukaryotic cells can express various proteasome isoforms. The most active isoforms of proteasomes are 26S proteasomes, which have a core (20S proteasome) coupled to 19S regulatory complex and process poly-ubiquitinated substrates^5^. 20S proteasomes, alone or coupled to other regulatory complexes such as PA28, can destroy proteins even in a ubiquitin-independent fashion^6^. The various human 20S proteasome isoforms are all constituted by two α and two β heptameric rings. Standard proteasomes (s-proteasomes) contain the catalytic β1, β2, and β5 subunits, whereas immunoproteasomes (i-proteasomes) contain the β1i, β2i, and β5i subunits. The latter are constitutively present in immune cells as well as in cells exposed to an inflammatory milieu. Intermediate-type and subtype proteasomes have been also described. Furthermore, a third proteasome isoform, *i.e*. the thymoproteasome, is expressed in thymic cortex and regulates T cell repertoire^4^. These proteasome isoforms have different proteolytic dynamics and sequence preferences, although it is still a matter of debate whether they generate a distinct set of peptides^7–14^.

**Fig. 1:**
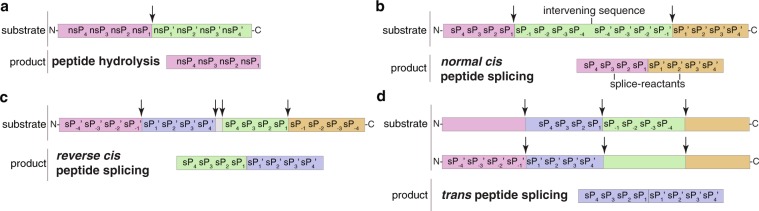
Proteasome-catalyzed peptide hydrolysis and peptide splicing. Proteasomes form peptide fragments by: (**a**) peptide hydrolysis and (**b,c**) *cis* peptide splicing, when the two splice-reactants derive from the same polypeptide molecule; peptide fragment ligation can occur in normal order, *i.e*. following the orientation from N- to C-terminus of the parental protein (normal *cis* peptide splicing; **b**), or in reverse order (reverse *cis* peptide splicing; **c**); (**d**) *trans* peptide splicing, when the two splice-reactants originate from two distinct molecules of the same protein or two distinct proteins. The two fragments, bound together during the peptide splicing reaction, are named splice-reactants. The portion between two splice-reactants is called intervening sequence. In this schematic, we summarize the residue positions (from nsP_4_ to nsP_4_’ for non-spliced peptides and from sP_4_ to sP_4_’ for spliced peptides) that were examined for the position frequency matrices, and which seem to be relevant in proteasome-catalyzed peptide hydrolysis and peptide splicing reactions. Arrows represent the substrate cleavage sites used by proteasome catalytic Thr1.

Proteasomes can cut proteins and release peptides by peptide hydrolysis, as well as recombine them by peptide splicing, thereby generating novel sequences, *i.e*. spliced peptides (Fig. 1a–d). Spliced peptides can originate from the ligation of two fragments of the same molecule (*cis* spliced peptides; Fig. 1b,c) or from two distinct molecules (*trans* spliced peptides; Fig. 1d). For both, proteasome-catalyzed peptide hydrolysis and peptide splicing, the residues surrounding the substrate cleavage and splicing sites seem to impinge upon the reaction efficiency^15–19^. Post-translationally spliced peptides represent a sizeable portion of HLA-I immunopeptidomes^20–23^ and of peptide products generated *in vitro* by proteasomes^17,19,24^.

Understanding which are the driving forces and the sequence preferences of both proteasome-catalyzed peptide hydrolysis and peptide splicing can streamline target discovery in novel immunotherapies. Indeed, both proteasome-generated spliced and non-spliced epitopes can trigger an immune response against cancer, infection and autoimmune-relevant antigens and this response can lead, for example, to tumor regression and to cure patients^25–30^. Predictors of proteasome-catalyzed peptide hydrolysis and peptide splicing could be integrated in some of the pipelines that have been proposed for a targeted epitope discovery, in the last years, within the framework of translational applications^24–26,31–36^. Various groups used *in vitro* 20S proteasome digestions, generally measured by mass spectrometry (MS), to define proteasome substrate preferences. Correspondence between *in vitro* experiments carried out with purified 20S proteasomes and *in cellulo* and *in vivo* experiments has been demonstrated in various studies investigating both viral and tumor epitopes^7–9,20,26,27,37–44^.

In the last two decades, few attempts have been done to predict proteasome-catalyzed peptide hydrolysis and to identify the first peptide splicing rules. Those attempts were all based on peptide product databases limited in size (both number of investigated substrates and detected products) and in sequence identification sensitivity, which could have impinged upon their predictive power^25,45–48^. Therefore, we here propose a large database containing spliced and non-spliced peptides produced *in vitro* by proteasome isoforms in different conditions, derived from 55 synthetic polypeptide substrates and measured with different MS equipment multiple times.

## Methods

### Peptide synthesis and proteasome purification

All peptides were synthesized using Fmoc solid phase chemistry. 20S s- and i-proteasomes were purified from peripheral blood (s-proteasomes), T2 cell line (s-proteasomes) or EBV-immortalized B lymphocytes (i-proteasomes) as follows: (i) either 10 ml human peripheral blood or 2 × 10^9^ cells were homogenized, lysed and centrifuged; (ii) the supernatant was fractionated by ammonium sulphate precipitation (35% and then 75%); (iii) the latter pellet was fractioned by chromatography on DEAE-Sephacel; (iv) the selected fractions were separated by 10–40% sucrose gradient and followed by (v) anion exchange chromatography on Mono Q in an Akta-FPLC system; (vi) the selected fractions (2–4 ml) were further purified by DEAE-Affi-gel-blue chromatography. In each of the (ii-vi) steps, the fractions were monitored by degradation assays of standard short fluorogenic substrate Suc-LLVY-AMC, which is a specific substrate for proteasome proteolytic activity^12^. Proteasome concentration was measured by Bradford staining and verified by Coomassie staining of an SDS-Page gel, as shown elsewhere^49^. The purity of the preparation using this protocol has been previously shown^13^. 20S and 26S proteasomes used in the digestions of the synthetic substrate TSN89 were purified from human erythrocytes and spleen, as previously described^50^. Both 20 i-proteasomes purified from human spleen and EBV-immortalized B lymphocytes contain mainly the catalytic β1i, β2i, and β5i subunits, although standard catalytic β1, β2, and β5 subunits are present too^13,51–53^, and have been considered as i-proteasomes in this study.

### *In vitro* digestions, MS measurements and analyses

Synthetic polypeptides were digested for different time points (0, 4, 20/24 h) at 37 °C by 20 S proteasomes in TEAD buffer (Tris 20 mM, EDTA 1 mM, NaN_3_ 1 mM, DTT 1 mM, pH 7.2) - as previously described^54^ - or by 20S/26S proteasomes in TSGD buffer (50 mM Tris/HCl pH 7.6, 10 mM KCL, 0.5 mM DTT, 5 mM MgCl, 2 mM ATP, 10% v/v glycerol) over time at 37 °C, as previously described^50^. The range of the ratio proteasome/substrate mildly varied from substrate to substrate to partially compensate the different degradation rates of substrates carried out by proteasomes. We used substrates with a final concentration of 40–80 μM and proteasome concentration of 1–5 μg per 100 μl reaction.

*In vitro* digestions were measured by the Shared Facility for Mass Spectrometry of the Charité (Berlin) using LTQ Orbitrap XL and Q Exactive Plus mass spectrometers, as well as by the Max Planck Institute for Biophysical Chemistry (MPI-BPC) Core Facility for Proteomics (Göttingen) using a Q Exactive Hybrid-Quadrupol-Orbitrap mass spectrometer.

In particular, the samples were measured by Charite’ facility by mean of nanoscale LC-MS/MS using an Ultimate 3000 and LTQ Orbitrap XL mass spectrometer (both Thermo Fisher Scientific) as follows: 10 µl digested polypeptide was loaded by an autosampler on a precolumn (PepMap C18, 5 mm × 300 μm × 5 μm, 100Ǻ, Thermo Fisher Scientific) with 2:98 (v/v) acetonitrile/water containing 0.1% (v/v) trifluoroacetic acid at a flow rate of 20 μl/min for 5 mins and separated by a 200 mm PicoFrit analytical column (PepMap C18, 3 µm, 100 Å, 75 µm; New Objective). A gradient 15–55% B in 90’ with a flow rate of 300 nl / min was used. The mobile phase (A) was 0.1% (v/v) formic acid in water, and (B) 80% acetonitrile in water containing 0.1% (v/v) formic acid. Full MS spectra (*m/z* 300–1,500) were acquired in an Orbitrap instrument at a resolution of 60,000 (FWHM). At first, the most abundant precursor ion was selected for collision-induced dissociation (CID) fragmentation (1^+^, 2^+^, 3^+^ charge state included) detected in an Ion Trap instrument. Additionally, the precursor ions were pre-elected for two Orbitrap CID- (resolution 7500) and higher-energy collisional dissociation (HCD*)* (resolution 15000) fragmentation scans. Dynamic exclusion was enabled with a repeat count of 2- and 30-s exclusion duration. The maximum ion accumulation time for MS scans was set to 200 ms and for MS/MS scans to 500 ms. Background ions at *m/z* 391.2843 and 445.1200 act as lock mass.

In addition, LC-MS/MS runs using a Q Exactive Plus mass spectrometer coupled with an Ultimate 3000 RSLCnano (Thermo Fisher Scientific) were performed as follows: samples were trapped as described above and then analyzed by the system that comprised a 250 mm nano LC column (Acclaim PepMap C18, 2 μm; 100 Å; 75 µm Thermo Fisher Scientific). Elution was carried out using a gradient 3–30% B in 90 min with the concentrations and conditions described above. The Q Exactive Plus instrument was operated in the data dependent mode to automatically switch between full scan MS and MS/MS acquisition. Full MS spectra (*m/z 200–*2,000) were acquired at a resolution of 70,000 (FWHM) followed by HCD MS/MS fragmentation of the top 10 precursor ions (resolution 17,500, 1^+^, 2^+^, 3^+^, charge state included, isolation window of 1.6 m/z, normalized collision energy of 27%). The ion injection time for MS scans was set to maximum 50 ms, Automatic Gain Control (AGCs) target value of 3 × 10^6^ ions and for MS/MS scans to 200 ms, AGCs 1 × 10^5^, dynamic exclusion was set to 20 s. The same ions were used as lock mass as described above.

For *in vitro* digestions measured by the MPI-BPC Core Facility for Proteomics (Göttingen), the following protocol was applied unless stated otherwise. Prior to measurement, the samples were diluted with the loading buffer (2% acetonitrile, 0.05% Trifluoroacetic acid) containing human insulin (Sigma-Aldrich) to a final substrate concentration of 25 μM and insulin concentration of 2 μM. Insulin was used as a coating polymer to prevent binding of peptides to the glass vials used for measurements and to improve reproducibility between technical replicates. 200 pmol of digestion sample (8 μl) were measured. Samples were separated by a nanoflow HPLC (RSLC Ultimate 3000) on an Easy-spray C18 nano column (30 cm length, 75 μm internal diameter; Dr. Maisch) coupled on-line to a nano-electrospray ionization Q Exactive Hybrid-Quadrupol-Orbitrap mass spectrometer (Thermo Fisher Scientific). Peptides were eluted with a linear gradient of 5%–55% buffer B (80% ACN, 0.1% formic acid) over 88 minutes at 50 °C at a flow rate of 300 nl/min. The instrument was programmed within Xcalibur 3.1.66.10 to acquire MS data in a Data Dependent Acquisition mode using Top 20 precursor ions. We acquired one full-scan MS spectrum at a resolution of 70,000 with an AGC target value of 1 × 10^6^ ions and a scan range of 350~1,600 m/z. The MS/MS fragmentation was conducted using HCD collision energy (30%) with an Orbitrap resolution of 35,000 at 2 m/z isolation window with Fixed First Mass set to 110 m/z. The AGC target value was set up at 1 × 10^5^ with a maximum injection time of 128 msec. For Data Dependent Scans the minimum AGC target value and the intensity threshold were set to 2,600 to 20,000 accordingly. A dynamic exclusion of 25 s and 1–6 included charged states were defined within this method. For the substrates TSN2, TSN45 and TSN93, no insulin was added and the samples were diluted with the loading buffer (2% acetonitrile, 0.05% Trifluoroacetic acid) to a final substrate concentration of 10 μM. 50 pmol of digested samples (5 μl) were measured employing the method described above.

Recalibrated tandem mass spectra were matched using Mascot with a mass tolerance of either 10 ppm (for XL mass spectrometer) or 6 ppm (for Q Exactive mass spectrometers) on precursor masses and either 0.5 Da for fragment ions using Ion Trap or 20 ppm using Orbitrap for fragmentation. Data have been searched against a custom database containing all theoretically possible spliced and non-spliced peptides derived from the substrate of interest. The following variable modifications have been set: NQ deamidation and M oxidation.

### Database

**Fig. 2:**
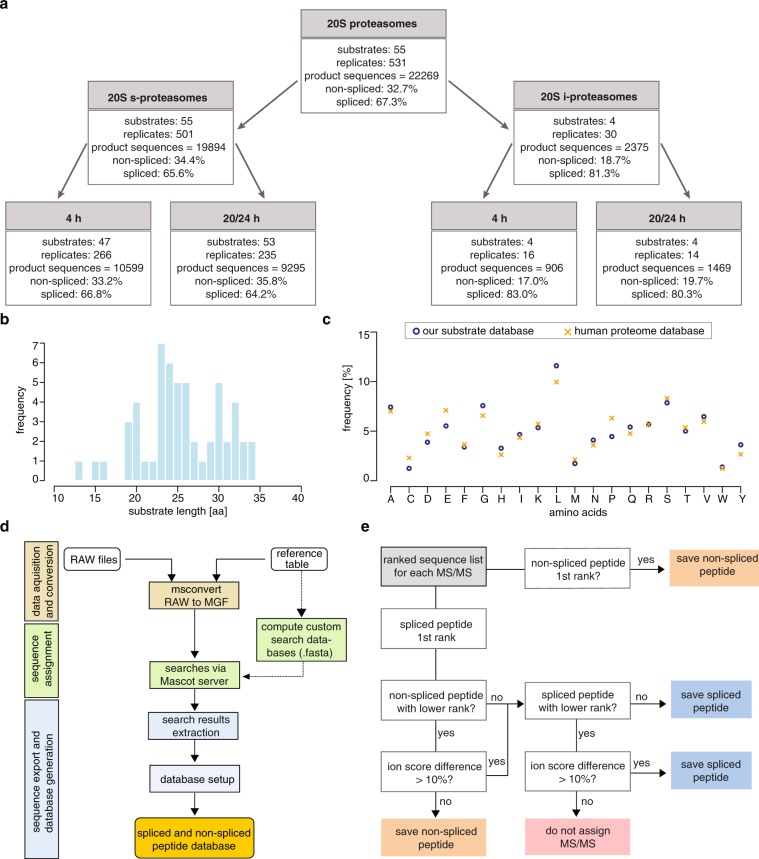
Database content and construction. (**a**) Database content. The database consists of peptide products identified in digestions of 55 unique substrates with 20 S and 26S s- and i-proteasomes at 4 h and 20/24 h digestion time. Shown is the main part, which represents all digestions with 20 S proteasomes. Values refer to product sequences (identified per sample). Several product sequences are identified in multiple experimental conditions using the sample substrate. Therefore, the number of unique peptide sequences, *i.e*. peptide sequences identified per substrate, is smaller than that of product sequences. (**b**) Length distribution of synthetic polypeptide substrates included in the database. (**c**) Matrixes of the amino acid frequency of the synthetic polypeptide substrates included in our database and of the human proteome database. (**d**) Overview of the data processing pipeline to construct the database. (**e**) Identification of spliced and non-spliced peptides from MS datasets, which was applied to assign peptides to each MS/MS spectrum.

Our database accounts for proteasome-mediated *in vitro* digestions of 55 unique synthetic polypeptide substrates (Fig. 2a). For each substrate we have carried out 1–4 biological replicates and each sample has been measured 1–5 times (*i.e.* technical replicates; median is 3 times).

Substrates vary from 13 to 34 amino acids long peptides (Fig. 2b). They have an amino acid frequency that is similar to that present in the human proteome (Fig. 2c). The polypeptides are a portion of bacterial, viral and human proteome proteins (largely antigens). Among the human antigens, there is a preponderant presence of tumor-associated or autoimmune disease-associated antigens. Biological origin of the substrate and unique identifier of the substrate sequences are attributes of our database (see also Table 1).

**Table 1:** Database description.

Column name	Description
sampleID	Unique identifier for every sample
sampleName	Sample Name used during experiment
runID	Technical replicate number
protIsotype	proteasome isoform used for digestion
digestTime	Elapsed digestion time (hours) at time of measurement
species	species origin for used proteasome
sampleDate	Sample date
instrument	Instrument used for measurement
fragmentation	Fragmentation method used for measurement
substrateOrigin	Biological origin of substrate
location	Measurement location
substrateSeq	Amino acid sequence of substrate
substrateID	Unique identifier for a substrate sequence
pepSeq	Amino acid sequence of peptide products
scanNum	Scan Number listed in the RAW file
rankMS	Peptide rank assigned by Mascot Server
ionScore	Ion Score assigned by Mascot Server
qValue	Q-Value assigned by Mascot Server
productType	PCP: non-spliced peptide; PSP: spliced peptide
spliceType	PSP: *cis*, reverse *cis* or *trans* spliced peptides
positions	Peptide sequence described as amino acid positions in the substrate sequence
charge	Ion charge
PTM	Post-translational modifications

Listed are the column names (*i.e*. Attributes) in the database and their corresponding explanations.

Experiments have been carried out with synthetic polypeptides rather than the entire protein because purified proteasomes hardly process entire proteins *in vitro*, likely because ligases and cofactors are lost during 20S/26S proteasome purification^55^. However, a correspondence between *in vitro* with purified proteasomes and *in vivo* experiments has been widely demonstrated (see text above).

Each digestion has been carried out with a single polypeptide as substrate. Hence, non-spliced and *cis* spliced peptides were produced by degradation of a single molecule of the substrate (Fig. 1a–c), whereas *trans* spliced peptides were the result of the ligation of two partially overlapping fragments derived from two molecules of the same substrate (Fig. 1d).

*In vitro* digestions have been performed at 0, 4 and 20/24 h. Peptide products identified only in the 4–20/24 h digestions have been included in the database, thereby removing synthesis artifacts (see Technical Validation section).

It is well known that the degradation rate of substrates varies from polypeptide substrate to substrate, from proteasome preparation to preparation. Therefore, based on our experience with the polypeptide substrates included in the database, we set the *in vitro* reaction conditions to have a time point – *i.e*. 4 h – when substrate molecules were still present in the reaction and another time point – *i.e*. 20/24 h – when all substrates molecules were, on average, (almost) completely processed. The presence of intact substrate molecules in the reaction can be determined by analyzing the MS RAW files linked to our database (see Data Record section).

*In vitro* digestions of 48 synthetic substrates, which represent the corner stone of our database, have been measured by XL Orbitrap (using both, Ion Trap and Orbitrap modes). *In vitro* digestions of 4 and 10 synthetic substrates have been measured also by Q Exactive Orbitrap at Charité Shared Facility for MS and by Q Exactive Orbitrap at MPI-BPC Core Facility for Proteomics, respectively.

*In vitro* digestions of 55 synthetic substrates have been carried out with 20S s-proteasomes. For four synthetic substrates, *in vitro* digestions have also been carried out with 20 S i-proteasomes. For one synthetic substrate, *in vitro* digestions have also been carried out with 20 S and 26S s- and i-proteasomes.

For seven substrate sequences, we performed *in vitro* digestions of both wild type and mutated synthetic polypeptides. A single residue mutation can, indeed, impinge upon proteasome dynamics, as shown for both tumor-associated and viral peptide sequences^24,38,54^.

To construct the database, we developed a computational pipeline that facilitates efficient and reproducible data processing. After MS RAW file collection, RAW files were renamed according to substrate ID, instrument type, proteasome isoform and digestion time points (to ensure a uniform data processing). The renamed RAW files were converted to the Mascot Generic Format (MGF) with ProteoWizard msconvert, using the vendor peak picking option. RAW files that contained XL Ion Trap and XL Orbitrap scans were split into separate files for each mass analyzer type. Afterwards, headers containing search parameters were added to the MGF files and submitted as MS/MS ion searches to the Mascot Server. Custom peptide databases containing all possible non-spliced and spliced peptides of a given substrate were computed and used, in the Mascot database searches, to assign peptide candidates to each MS/MS spectrum, *i.e*. peptide spectrum matches (PSMs). Search parameters including precursor and MS/MS mass tolerances were the same as described above. The search results were exported as CSV files and combined with additional sample information of each digestion and MS run to generate a preliminary database housing all peptide hits suggested by the Mascot Server (Fig. 2d). This preliminary database was then used to filter out wrong PSMs from likely correct PSMs (see below, Fig. 2e).

The final database output is a CSV table, which contains 23 columns describing features of the identified peptides, the original substrate sequence, sample processing and instrument parameters (see Table 1 for a detailed description of the database columns/attributes). Additionally, to the CSV file, we provide an SQL dump file, which can be directly loaded to a user SQL server.

### Generation and analysis of the random control dataset

In order to evaluate the sequence characteristics of the identified peptides in the database, we generated a random control dataset. Latter is generated by computing all theoretically possible spliced and non-spliced peptides according to Liepe *et al*.^56^, but without restrictions of intervening sequence length, splice-reactant length or product length. To note, this is the same sequence database which was employed to search the respective *in vitro* MS data.

In order to account for potential MS biases such as the MS detectable mass range used in the MS measurements (from 200 to 2000 m/z), we next removed all theoretical possible sequences, which have a length shorter than 6 amino acids and do not exceed a molecular weight of 7 kDa. Latter is set by determining the maximum molecular weights of all identified sequences. Due to the large number of theoretically possible spliced and non-spliced peptides, we generated a random peptide sample from each product type (*i.e*. non-spliced, normal *cis* spliced, reverse *cis* spliced and t*rans* spliced random peptide sample). The sample size was chosen according to the number of identified product types included in the database. When analyzing this random control dataset for sequence characteristics (sequence length and amino acid distributions), a non-uniform distribution of all characteristics is expected due to the length restriction and amino acid distribution of the 55 synthetic polypeptides. This means that if proteasomes -catalyzed peptide hydrolysis and peptide splicing were random processes, *i.e*. not following any specificities, we would expect to observe length distributions and amino acid distributions identical to those of the random control dataset. We therefore compared the observed length distributions of identified products, splice-reactants and intervening sequences to those of the random control dataset (see Technical Validation section).

### Analysis of amino acid content in substrates and products

The amino acid distribution of the synthetic polypeptide substrates included in the database follows that of the human reference proteome (Fig. 2c), and clearly diverts from a uniform distribution. Furthermore, the amino acid distribution within a given synthetic polypeptide is not uniform. Such structure impinges on the analysis of amino acid distributions of identified peptide products. In order to account for those structure -related effects of the substrates, we computed the amino acid frequencies of the peptide products identified in 20/24 h digestions as well as the amino acid frequencies of the random control dataset. The amino acid frequencies of the random control sample represent the structure of the synthetic polypeptides. We next normalize the frequencies of the peptide products identified in 20/24 h digestions with those frequencies obtained from the random control dataset. Similarly, the amino acid frequencies of the detected synthesis artefacts were also normalized. The resulting amino acid frequencies are discussed in the Technical Validation section.

### Statistical analysis

If not stated otherwise, all statistical tests have been done in R. Differences in distributions have been tested using the Kolmogorov-Smirnov test. Where appropriate, p-values have been adjusted with Bonferroni correction.

## Data Records

The MS files on which the database has been built have been deposited to the PRIDE repository with the dataset identifier PXD016782^57^. The final database with all identified spliced and non-spliced peptide products, as well as their substrate sequences, is provided as a simple CSV file as well as a MySQL database and has been deposited to the Mendeley repository with the dataset access doi:10.17632/nr7cs764rc.1^58^.

## Technical Validation

### MS-based identification of high-confidence spliced and non-spliced peptides

Spliced peptide identification could be biased by the large number of theoretically possible spliced peptides derived from one substrate in the database and by the presence of synthesis artifacts^24^. Therefore, we adopted a stringent pipeline for spliced peptide identification and the generation of a high-quality database. We used the preliminary database containing all PSMs - including all ranks - suggested by the Mascot Server as the basis for a conservative filtering approach that aimed to identify high-confidence spliced and non-spliced peptides (Fig. 2e). For this, all ranked PSMs suggested by the Mascot Server for a single MS/MS scan (query) have been evaluated based on peptide type (spliced vs non-spliced) and differences in ion scores to determine the most probable peptide sequence and origin. Scans that did not allow for the high-confidence identification of a single peptide were not assigned and removed from further analysis. In the case that the top ranked peptide was non-spliced, it was considered a correct PSM if the Mascot ion score was higher than 20 and the Mascot q-value was lower than 0.05. However, in the case that the top ranked peptide was a spliced peptide, further steps were taken to ensure a correct identification. If multiple top ranked spliced peptides were identified, the whole scan was discarded, because the correct sequence could not be determined from the information in the MS/MS scan. If only a single top ranked spliced peptide was suggested by Mascot, the second ranked peptide was considered for comparison and evaluated based on the difference in the ion score between top ranked and lower ranked peptides. A difference of less than 10% between a top-ranked spliced and a lower ranked non-spliced peptide led to the assignment of the non-spliced peptide as most likely correct PSM. If no suggested lower ranked non-spliced peptide was present, we calculated the difference in ion scores between the top ranked spliced peptide and any lower ranked spliced peptide. The top-ranked spliced peptide was only assigned as correct PSM if it had an ion score at least 10% larger than any lower ranked peptide. If latter was not the case, the query was not assigned and removed from further analysis. Finally, all suggested spliced and non-spliced peptides must have had an ion score larger than 20 and a q-value lower than 0.05 (Fig. 2d).

This approach favors the assignment of non-spliced peptides over spliced peptides to counteract the imbalance of the number of spliced and non-spliced peptide sequences in the MS search space.

Spliced peptides generated by ligation of three or more fragments were not allowed and therefore are not in our database.

Synthesis artifacts are an unwanted but inherent factor of any experiment with synthetic polypeptides. The MS equipment used in this study, indeed, is so sensitive that it can detect even small amounts of artifacts generated during the synthesis of the polypeptide sequences. To avoid the inclusion of synthesis artifacts in our database, we measured each substrate either at 0 h digestion time or in absence of proteasomes. Each peptide identified in those samples represents a synthesis error and therefore should not be considered as proteasome-generated peptide. We used the peptide sequences identified in those control measurements to remove any non-spliced peptide that showed the same sequence at later digestion time points, as well as any spliced peptides that showed the same sequence or a subsequence at later digestion time points.

The correct identification of some of the spliced and non-spliced peptides included in our database has already been demonstrated elsewhere by measuring digestion samples and synthetic peptides with the same method and comparing MS/MS and retention time of the peptides^22–24^.

**Fig. 3:**
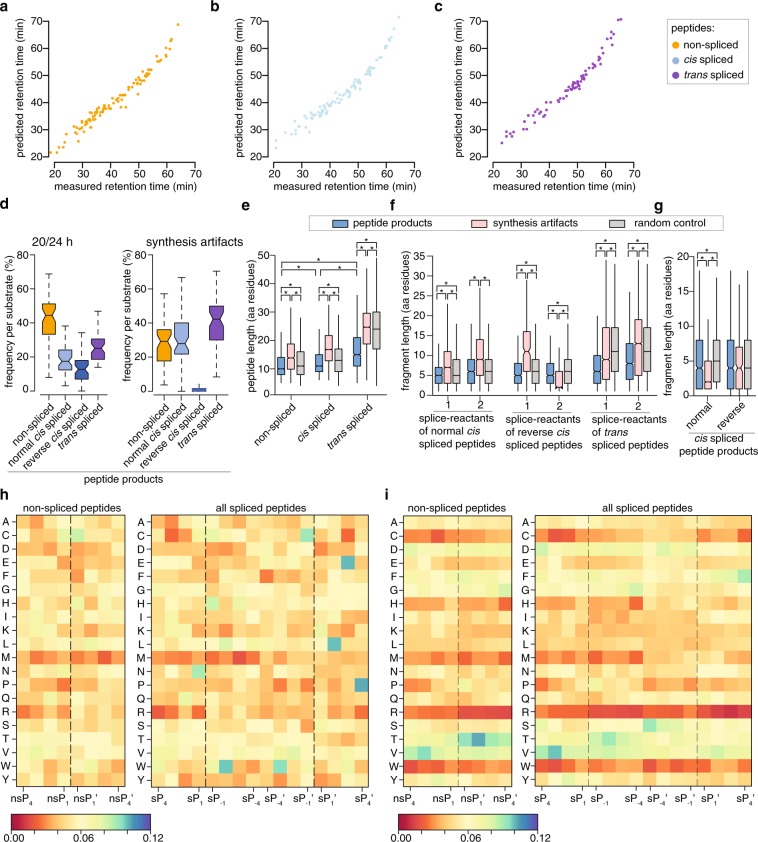
Database validations and characteristics of spliced and non-spliced peptides products. (**a-c**) Comparison of measured and predicted retention time of non-spliced, *cis* and *trans* spliced peptides identified in our database. Non-spliced peptides were used to train a retention time model (**a**), which was then used to predict the retention times of identified *cis* spliced (**b**) and *trans* spliced peptides (**c**). (**d,e**) Relative frequency (**d**) and length distribution (**e**) of non-spliced, *cis* spliced and *trans* spliced unique peptides generated after 20/24 h digestion by 20S s-proteasomes. This analysis is done on unique peptide sequences (*i.e*. unique sequences identified per substrate). For database validation, length distribution of synthesis artifacts (**d,e**) and of random control dataset (**e**) are shown. (**f,g**) Length distribution of N-terminal (splice-reactant 1) and C-terminal (splice-reactant 2) splice-reactants (**f**) and intervening sequence length distribution (**g**) of spliced peptide products detected in 20/24 h *in vitro* digestions with 20S s-proteasomes are shown. As comparison, length distribution of synthesis artifacts and of random control dataset are shown. Statistically significant comparisons are labeled with * and the related p values are reported in Table 2. (**h,i**) Matrixes of the amino acid frequency, in the position enumerated in Fig. 1, of non-spliced and spliced peptide products generated by 20S s-proteasomes after 20/24 h (**h**) and synthesis artifacts identified in control samples (**i**).

To further validate the identified non-spliced, *cis* and *trans* spliced peptides, we predicted the retention time of the identified peptides and compared them to the measured retention times by MS^59,60^. The identification of non-spliced peptides in *in vitro* digestions is straightforward. Therefore, we used the measured retention times of non-spliced peptides to train a dataset-specific retention time model. The latter was used to predict retention times of detected spliced peptides. The relation between measured and predicted retention times of spliced peptides should reflect the same behavior as those for non-spliced peptides if the identification of sequences were correct (Fig. 3a–c). A similar approach was used in our SPI-delta method to filter spliced peptide candidates in HLA-I immunopeptidomes^23^.

### Spliced and non-spliced peptide analysis and further validations

Our database accounts for 22,333 product sequences (7,305 non-spliced, 7,323 *cis* spliced and 7,705 *trans* spliced product sequences). Several product sequences are identified in more than one sample of the same substrate. Therefore, the number of unique peptide sequence, *i.e*. peptide sequences identified per substrate, is smaller that that of product sequences. In particular, our database contains 14,433 unique sequences (3,834 non-spliced, 5,011 *cis* spliced and 5,588 *trans* spliced unique peptide sequences). *Cis* and *trans* spliced unique peptide sequences represent 34.7% and 38.7%, respectively, of the unique peptide sequences present in our database.

To note, spliced peptides were identified after applying the stringent filter strategy for spliced peptide identification (described above) and the removal of any potential synthesis artifact, which might have eliminated spliced peptides that were actually produced by proteasomes.

Furthermore, the similar frequency of spliced product sequences identified in 4 h and 20/24 h digestions catalyzed by either 20S s- and i-proteasomes (Fig. 2a) confirmed that in our experimental conditions the large frequency of spliced peptides was not due to an unphysiological re-entry of the products coupled to a prevalent ligation of peptide fragments.

As proof of principle, we performed a first analysis of the features of peptide products identified in the digestions of 53 synthetic substrates with 20S s-proteasome after 20/24 h. In this database subset, we identified 9,295 products sequences (35.8% non-spliced and 64.2% spliced peptide product sequences; Fig. 2a), which correspond to 8,651 unique peptide sequences (3,049 non-spliced, 3,038 *cis* spliced and 2,564 *trans* spliced unique sequences). When we analyze the unique peptide sequences identified in this experimental condition, we observe that the relative frequency of spliced vs non-spliced peptides varies largely among substrates (Fig. 3d). There are significant differences in the length distribution of non-spliced vs *cis* spliced vs *trans* spliced peptide products per substrate. Among them, *trans* spliced peptides are the longest, as expected bearing in mind that they can (as it happened) be even longer than the original synthetic substrate. *Cis* spliced peptides are significantly longer than non-spliced peptides (Fig. 3e).

To further validate our database, we computed the distributions of the peptide length for all peptides identified in substrate controls, which are synthesis artifacts (Fig. 3d–g). Among the synthesis artifacts we annotated non-spliced and spliced peptides. We identified only very few reverse *cis* spliced peptides among those artifacts, as expected, considering the protocols of peptide synthesis (Fig. 3d). As mentioned earlier, all synthesis artifacts and potentially derived sequences have been removed from the final database. Therefore, synthesis artifacts represent a useful negative control database. The length distributions of synthesis artifacts, of their splice-reactants and intervening sequences are significantly different than those of identified peptide products (but the splice-reactant 2 of reverse *cis* spliced peptide of synthetic artifacts; see Fig. 3e–g and Table 2). This suggests that the spliced peptides of our database are not artifacts.

**Table 2:** Tests for statistical differences between characteristics of identified peptides.

Group 1	Group2	p-value
***Peptide length***		
Non-spliced products	Non-spliced synthesis artifacts	<2e-16
Non-spliced products	Non-spliced random control	<2e-16
Non-spliced synthesis artifacts	Non-spliced random control	<2e-16
*cis* spliced products	*cis* spliced synthesis artifacts	<2e-16
*cis* spliced products	*cis* spliced random control	<2e-16
*cis* spliced synthesis artifacts	*cis* spliced random control	<2e-16
*trans* spliced products	*trans* spliced synthesis artifacts	<2e-16
*trans* spliced products	*trans* spliced random control	<2e-16
*trans* spliced synthesis artifacts	*trans* spliced random control	<2e-16
Non-spliced products	*cis* spliced products	<2e-16
*cis* spliced products	*trans* spliced products	<2e-16
Non-spliced products	*trans* spliced products	<2e-16
***Splice-reactant length: SR1***		
Normal *cis* spliced products	Normal *cis* spliced synthesis artifacts	<2e-16
Normal *cis* spliced products	Normal *cis* spliced random control	<2e-16
Normal *cis* spliced synthesis artifacts	Normal *cis* spliced random control	<2e-16
Reverse *cis* spliced products	Reverse *cis* spliced synthesis artifacts	<2e-16
Reverse *cis* spliced products	Reverse *cis* spliced random control	6.3e-16
Reverse *cis* spliced synthesis artifacts	Reverse *cis* spliced random control	7.9e-13
*trans* spliced products	*trans* spliced synthesis artifacts	<2e-16
*trans* spliced products	*trans* spliced random control	<2e-16
*trans* spliced synthesis artifacts	*trans* spliced random control	1.8e-11
***Splice-reactant length: SR2***		
Normal *cis* spliced products	Normal *cis* spliced synthesis artifacts	<2e-16
Normal *cis* spliced products	Normal *cis* spliced random control	0.055
Normal *cis* spliced synthesis artifacts	Normal *cis* spliced random control	<2e-16
Reverse *cis* spliced products	Reverse *cis* spliced synthesis artifacts	<2e-16
Reverse *cis* spliced products	Reverse *cis* spliced random control	<2e-16
Reverse *cis* spliced synthesis artifacts	Reverse *cis* spliced random control	<2e-16
*trans* spliced products	*trans* spliced synthesis artifacts	<2e-16
*trans* spliced products	*trans* spliced random control	<2e-16
*trans* spliced synthesis artifacts	*trans* spliced random control	<2e-16
***Intervening sequence length***		
Normal *cis* spliced products	Normal *cis* spliced synthesis artifacts	0.013
Normal *cis* spliced products	Normal *cis* spliced random control	<2e-16
Normal *cis* spliced synthesis artifacts	Normal *cis* spliced random control	<2e-16
Reverse *cis* spliced products	Reverse *cis* spliced synthesis artifacts	0.042
Reverse *cis* spliced products	Reverse *cis* spliced random control	0.014
Reverse *cis* spliced synthesis artifacts	Reverse *cis* spliced random control	0.14

Group 1 is compared to group 2 using Kolmogorov-Smirnov test. Resulting p-values are listed.

As further database validation, we generated a random control dataset consisting of a random sample of any potential spliced and non-spliced peptide that could be derived from the analyzed substrate sequences (see also “Generation and analysis of the random control dataset” section). If proteasome-catalyzed peptide splicing was not a random process, but followed specific rules, we would expect to observe significant difference in sequence characteristics through the comparison of random controls and the identified peptide products. Accordingly, identified spliced peptide products have a statistically significant different distribution of their length, as well as the length of splice-reactants and intervening sequences as compared to the random control dataset – but the reverse *cis* spliced peptides (Fig. 3f,g and Table 2). These comparisons further confirm that the spliced and non-spliced peptide products identified with our method, and reported in the database, are not artifacts and have been correctly identified.

As last database validation, in the 20/24 h *in vitro* digestion with 20S s-proteasomes and in the synthetic artifacts databases, we calculated the frequency of amino acids in the positions surrounding the substrate cleavage sites theoretically used to generate splice-reactants and non-spliced peptides (see Fig. 1 for enumeration). To account for the amino acid frequency in the synthetic substrates included in the database, we normalized the position frequency matrixes for non-spliced and spliced peptide products (Fig. 3h) and synthesis artifacts (Fig. 3i) by the amino acid frequency resulting from the random control database (see also “Analysis of amino acid content in substrates and products” section). The comparison of the matrixes of the peptide products and the synthesis artifacts is clearly discordant, which represent our last validation controls (Fig. 3h,i and Table 2).

Regarding the amino acid frequencies of peptide products, a preliminary comparison suggests that 20S s-proteasomes prefer amino acids with polar uncharged side chains (S, T, N, Q) as well as small amino acids (A, G) in position nsP1 and sP1 to catalyze peptide hydrolysis and splicing, respectively, and hydrophobic amino acids seem to be favored in position sP1’ of spliced peptides. However, both peptide hydrolysis and splicing show complex amino acid preferences, which can be investigated in depth with our database and used for the development of algorithms predicting both peptide hydrolysis and splicing.

As last note, the frequency of spliced peptides in our database is strongly larger than what we previously estimated in similar experimental conditions^19^. Such a discrepancy can be explained through the improvement of MS sensitivity and accuracy in the last decade. For example, with the older generation Thermo Fisher Deca MS previously used, we identified 35 non-spliced and 12 spliced peptide products in *in vitro* digestions of the substrate TSN2^19^. In experiments carried out in similar conditions, measured with Orbitrap Q Exactive mass spectrometer (MPI-BPC Göttingen), we identified 53 non-spliced peptides and 99 spliced peptides, which are included in our database.

However, it is worth to mention that the frequency values reported in this manuscript refer to peptide variety and not peptide amount. We speculate that the amount – *i.e*. number of molecules - of spliced peptides is smaller than non-spliced peptides in the *in vitro* digestions included in this study, in agreement with other studies^19,24^.

## Usage Notes

The database is provided as CSV file, which can be opened in Excel or any text editor, as well as MySQL database dump for more convenient downstream analysis. Both files have been deposited to the Mendeley repository with the dataset access doi:10.17632/nr7cs764rc.1^58^.

## Data Availability

The algorithm generating all possible *cis* and *trans* spliced peptides was originally described by Liepe *et al*.^56^. Scripts for MySQL database setup have been deposited in the MySQL database dump to the Mendeley repository with the dataset access doi:10.17632/nr7cs764rc.1^58^.
